# Supplementary material for: Cannabinoids Activate Endoplasmic Reticulum Stress Response and Promote the Death of Avian Retinal Müller Cells in Culture
Source: Brain Sci. 2025 Mar 10;15(3):291. doi: 10.3390/brainsci15030291 (PMC11940308; doi:10.3390/brainsci15030291)
Supplement: Supplementary file 1 [file brainsci-15-00291-s001.zip › brainsci-3403721-supplementary.pdf]

Supplementary figure S1

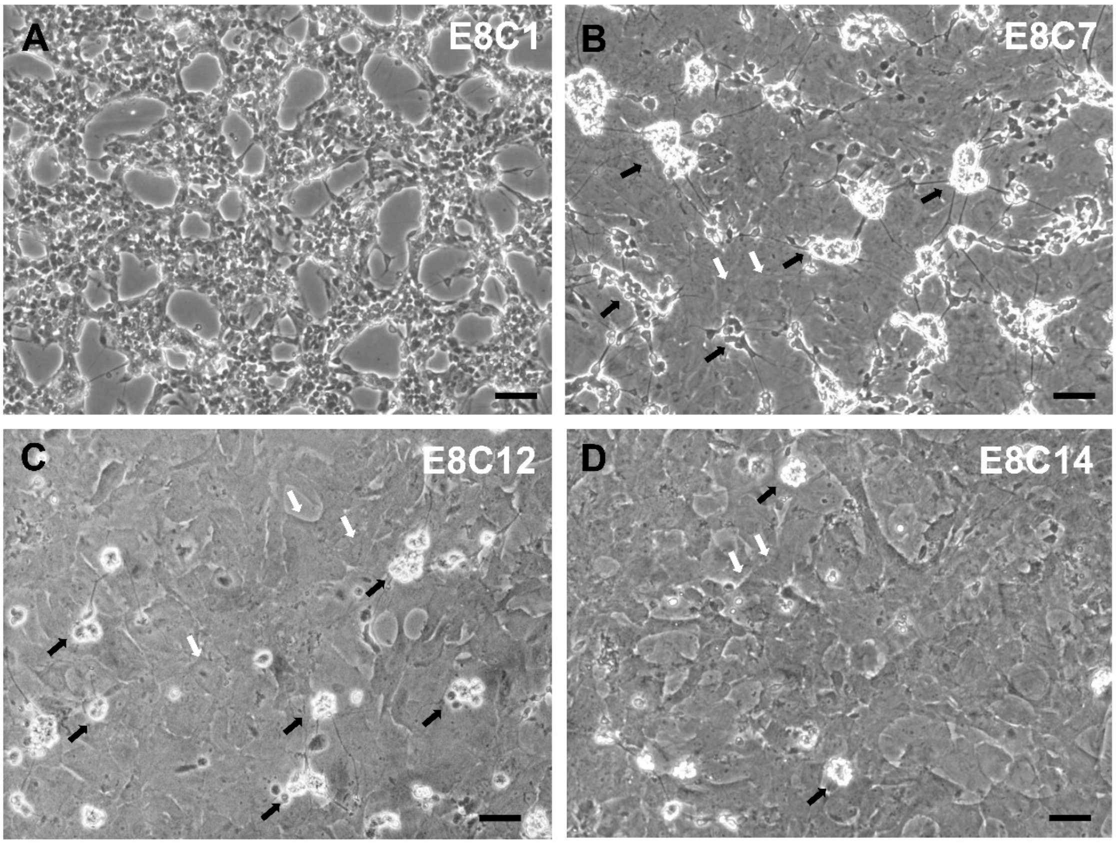

Figure S1. Development of the retinal cell monolayer cultures as a function of the cultivation time. Cells of retinas from 8-day-old chick embryos (E8) were seeded on plastic dishes and cultivated for several periods of time (E8C1 to E8C14). Cultures were photographed under phase contrast illumination and representative micrographs are shown. (A) E8C1, (B) E8C7, (C) E8C12, (D) E8C14. Note the progressive appearance of clusters of neuronal cells (black arrows) with the concomitant proliferation of attached glial cells that become flat, with expanded cytoplasm (white arrows). As the culture develops, neuronal clusters detach from the glial “carpet” and cultures becomes enriched in glial cells. Scale bar = 50 µm.
